# Early neurological deterioration in Wilson’s disease: a systematic literature review and meta-analysis

**DOI:** 10.1007/s10072-023-06895-6

**Published:** 2023-06-14

**Authors:** Agnieszka Antos, Anna Członkowska, Lukasz Smolinski, Jan Bembenek, Adam Przybyłkowski, Marta Skowrońska, Iwona Kurkowska-Jastrzębska, Tomasz Litwin

**Affiliations:** 1 Second Department of Neurology, Institute of Psychiatry and Neurology, Sobieskiego 9, 02-957 Warsaw, Poland; 2 Department of Gastroenterology and Internal Medicine, Medical University of Warsaw, Warsaw, Poland; 3 Department of Clinical Neurophysiology, Institute of Psychiatry and Neurology, Warsaw, Poland

**Keywords:** Wilson’s disease, Magnetic resonance imaging, UWDRS, Neurological deterioration, Chelators, Zinc salts

## Abstract

**Introduction:**

Neurological deterioration, soon after anti-copper treatment initiation, is problematic in the management of Wilson’s disease (WD) and yet reports in the literature are limited. The aim of our study was to systematically assess the data according to early neurological deteriorations in WD, its outcome and risk factors.

**Methods:**

Using PRISMA guidelines, a systematic review of available data on early neurological deteriorations was performed by searching the PubMed database and reference lists. Random effects meta-analytic models summarized cases of neurological deterioration by disease phenotype.

**Results:**

Across the 32 included articles, 217 cases of early neurological deterioration occurred in 1512 WD patients (frequency 14.3%), most commonly in patients with neurological WD (21.8%; 167/763), rarely in hepatic disease (1.3%; 5/377), and with no cases among asymptomatic individuals. Most neurological deterioration occurred in patients treated with d-penicillamine (70.5%; 153/217), trientine (14.2%; 31/217) or zinc salts (6.9%; 15/217); the data did not allow to determine if that reflects how often treatments were chosen as first line therapy or if the risk of deterioration differed with therapy. Symptoms completely resolved in 24.2% of patients (31/128), resolved partially in 27.3% (35/128), did not improve in 39.8% (51/128), with 11 patients lost to follow-up.

**Conclusions:**

Given its occurrence in up to 21.8% of patients with neurological WD in this meta-analysis of small studies, there is a need for further investigations to distinguish the natural time course of WD from treatment-related early deterioration and to develop a standard definition for treatment-induced effects.

**Supplementary information:**

The online version contains supplementary material available at 10.1007/s10072-023-06895-6.

## Introduction

Wilson’s disease (WD) is an inherited disorder of copper metabolism with pathological copper accumulation, which can result in clinical symptoms in affected organs, particularly hepatic and/or neuropsychiatric manifestations [1–4]. As the disease is caused by copper overload, WD treatment is based on drugs promoting negative copper balance including (1) copper chelators (d-penicillamine [DPA], trientine [TN] or dimercaptopropane sulfonic acid [DMPS] used in China) which mainly increase urinary copper excretion; (2) drugs which inhibit copper absorption from the digestive tract (zinc salts [ZS]); or (3) drugs complexing copper into insoluble complexes, leading to increase biliary copper excretion, as well as decrease copper absorption (e.g. molybdenum salts in clinical trials) [5].

WD can be successfully treated with pharmacological agents if diagnosis is established early and anti-copper treatment is correctly introduced and monitored [5–8]. Based on international WD registries, a satisfactory outcome is reached in almost 85% of WD patients [9, 10]. Improvement of hepatic symptoms as well as liver function tests usually occurs within the first 2–6 months of anti-copper treatment initiation [1]. Improvement of neurological symptoms may take longer and in some cases, can be observed up to 3 years after treatment initiation [1]. However, since DPA was introduced in 1956, the devastating phenomenon of early (so-called ‘paradoxical’) neurological deterioration after treatment initiation has been described in some patients [11–26].

Two types of clinical neurological deterioration during treatment can be distinguished: (1) early worsening that usually occurs up to 6 months of treatment initiation and is mostly connected with anti-copper therapy as a trigger and (2) late worsening, observed after 6 months of treatment introduction, which mainly results from non-compliance with anti-copper treatment and other factors [4, 5]. The majority of published cases of early neurological deterioration occurred in patients with the neurological phenotype and sometimes followed a dramatic course, for example, introduction of a full dose of DPA that led to the patient worsening to a bedridden state, which was frequently irreversible [15–17, 19, 24]. Over time, an association was made between introduction of the full dose of DPA and neurological deterioration [15–17, 19, 24] and a slow DPA titration scheme was introduced, which decreased the number of cases of early neurological deterioration [1, 5, 10]. However, the problem of early neurological deterioration has been reported after the introduction of all available anti-copper medications (DPA, TN, ZS, molybdenum salts) with different results [27–44], suggesting that alternative explanations for this phenomenon need to be taken into account [27–45].

Despite acknowledgement of the severe and even life-threatening consequences of early neurological deterioration, which is also a problem in the development of new WD treatments, there remains a lack of comprehensive studies on frequency and predictors. Numerous retrospective analyses of large national WD cohorts have assessed the frequency of neurological deterioration as part of secondary analyses; however, most did not use objective neurological scales [6–8]. Only a small number of studies and case reports have specifically analysed the phenomenon and investigated predictors [27, 28, 35, 43].

The aim of our study was to perform a systematic review and meta-analysis of published studies on early neurological deterioration, as well as discuss its definition and summarize risk factors.

## Methods

This systematic review was performed in concordance with internationally accepted criteria of the Preferred Reporting Items for Systematic Reviews and Meta-analyses (PRISMA) statement [46].

### Search strategy and eligibility criteria

We searched the PubMed database (up to 15 September 2022) for original studies (prospective and retrospective), as well as case and series reports analyzing early neurological deterioration, its frequency, risk factors and outcomes in patients with WD. Search terms included: (“Wilson’s disease”/ “Wilson disease” and “early neurological worsening”), (“Wilson disease”/ “Wilson disease” and “early neurological deterioration”), (“Wilson’s disease”/ “Wilson disease” and “neurological deterioration”) and (“Wilson’s disease”/ “Wilson disease” and “neurological worsening”). Studies eligible for further analysis were (1) human studies; (2) original studies (prospective or retrospective); (3) case and series reports of pregnant WD patients; and (4) those published in English. The reference lists of extracted publications were also searched. For the purpose of this study, we defined neurological deterioration as worsening in the 6-month period after treatment introduction.

Initially, the titles and abstracts of papers retrieved by the search terms were screened independently by all authors and duplicate records were removed. Reviews, editorial, commentaries, as well as overlapped studies were excluded after assessment and revisions. Then the full text of initially eligible articles was screened. Again, incomplete reports, reviews, editorials, commentaries, conference proceedings, discussions and overlapped studies were excluded. Finally, all identified studies were analyzed and verified independently by all authors to confirm the inclusion criteria and were grouped as (1) prospective studies that aimed to analyze early neurological deterioration; (2) retrospective studies mostly presenting data from country WD registries with additional analyses of early neurological deterioration; (3) retrospective analyses focused on the early neurological deterioration phenomenon; 4) series reports and (5) case reports describing patients who deteriorated shortly after decoppering treatment initiation; and 6) diagnosis of early neurological deterioration established based on objective neurological examination (preferably using neurological scales). Only WD drug naive patients were analyzed, also the analysis according to WD phenotypic presentation if available was summarized separately. For each publication, the number of patients involved, the definition of early neurological deterioration used (if any), the phenotype of WD, the risk factors of deterioration, the treatment type and the outcome were recorded, where available.

### Meta-analysis methodology

A random effects meta-analysis was used to estimate the risk of an early neurological deterioration among patients with the neurological form at diagnosis because deteriorations after treatment initiation almost never occur in other patients with WD. Heterogeneity was assessed with I2 and the chi-squared test for the Cochrane’s Q statistic. The Baujat plot was used to find studies with the greatest contribution to heterogeneity; a sensitivity analysis was carried out after the exclusion of these studies.

The study protocol was registered and received INPALSY registration number: INPALSY202290111 (https://doi.org/10.37766/inpalsy2022.9.0111).

## Results

**Fig. 1:**
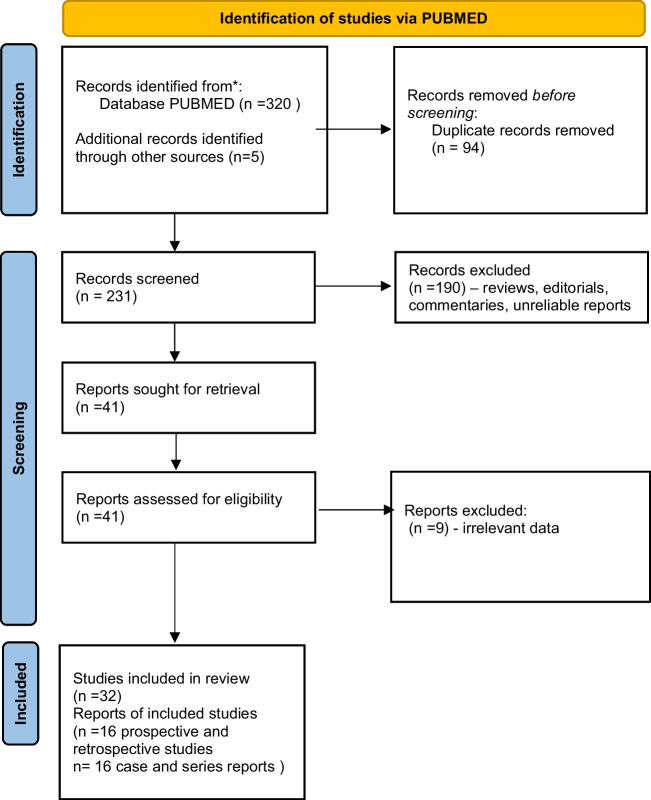
Flow chart of the systematic literature search according to PRISMA guidelines. A total of 320 articles were found during the initial screen and 32 articles were included in the qualitative synthesis

The publications search and selection process is presented in Fig. 1.

The initial PubMed search retrieved 320 records. During the search of extracted lists of references, we found an additional 5 papers [15, 25, 47–49]. After removal of duplicate articles, 231 publications remained. Titles, abstracts and full texts were then screened for relevance. Finally, 32 articles were included in the analysis. There were 7 prospective [32, 34, 39–41, 43, 44] and 9 retrospective studies [27, 28, 30, 33, 35–38, 42] presenting the early neurological deteriorations in WD patients (Table 1). There were also 16 case reports and case series presenting detailed descriptions of WD patients who had early neurological deterioration [12, 13, 15–20, 22–26, 47–49] (Table 2).

**Table 1:** Summary of studies analyzing the early neurological deterioration in WD patients according to their frequency, initial phenotype of WD patients, anti-copper treatment, potential risk factors of neurological deterioration as well as outcome of deterioration

Reference	Study details and definition of early neurological deterioration	Frequency of early neurological deterioration overall and by WD phenotype	Type of WD treatment in deteriorated patients and any information on risk factors for deterioration	Outcome after neurological deterioration
Ziemssen et al. [27]	Retrospective analysis of 61 patients Definition: any increase in UWDRS part II or an increase of ≥ 4 in part III or appearance of new neurological signs	Frequency of early neurological deterioration: Overall: 16.3% (10/61) In neurological WD: 27.7% (10/36) In hepatic WD: 0% (0/18) In presymptomatic individuals: 0% (0/7)	Type of treatment in deteriorated patients: DPA: 60% (6/10) Zinc sulfate: 40% (4/10) (NS vs DPA) Risk factors of early neurological deterioration: - Initial sNfL concentrations - Initial severity of neurological WD scored in UWDRS part II and III - Brain MRI chronic damage score scored in semiquantitative brain MRI WD scale	Not provided
Hou et al. [28]	Retrospective analysis of 47 patients Definition: Increase > 2 points in modified Young scale	Frequency of early neurological deterioration: In neurological WD (all patients): 61.7% (29/47)	Type of treatment in deteriorated patients: DPA: 100% (29/29) One patient discontinued DPA due to fever and rash and received DMPS without worsening Risk factors of early neurological deterioration: - Younger age at symptoms onset - Shorter delay of diagnosis - More frequently dystonic symptoms - Severe mutation in ATP7B gene type (frameshift; splicing; nonsense)	Persistent symptoms: 20.7% Partial recovery: 55.2% Recovery: 24.1%
Samanci et al. [33]	Retrospective analysis of 53 patients Definition: Not provided	Frequency of early neurological deterioration: In neurological WD (all patients): 20.7% (11/53)	Type of treatment in deteriorated patients: DPA: 72.7% (8/11) TN: 27.2% (3/11) Risk factors of early neurological deterioration: Not analyzed	Persistent: 36.3% (4/11) Partial recovery: 27.2% (3/11) Lost to follow-up: 36.3% (4/11)
Zhang et al. [30]	Retrospective study of 158 patients Definition: Any increase in GAS for WD or decrease in Barthel index	Frequency of early neurological deterioration: In neurological WD (all patients): 20.8% (33/158)	Type of treatment in deteriorated patients: DPA: 33.8% (22/65) DMPS and zinc acetate: 11.8% (11/93) Risk factors of early neurological deterioration: Not analyzed	Persistent: 84.8% (28/33) Recovery: 15.1% (5/33)
De Fabregues et al. [34]	Prospective study of 5 patients (only 2 drug-naive) Definition: Not provided (assessed by GAS for WD, UWDRS and Brewer-adapted UHDRS)	Frequency of early neurological deterioration: In neurological WD (all patients): 0% (0/2)	Type of treatment in deteriorated patients: Ammonium tetrathiomolybdate: 2/2 Risk factors of early neurological deterioration: Not analyzed	Not applicable
Ranjan et al. [32]	Prospective study of 34 patients Definition: Any increase in neurological severity grade (I–IV) or in Burke-Fahn-Marsden score (dystonia) or decrease in Barthel index	Frequency of early neurological deterioration: In neurological WD (all patients): 17.6% (6/34)	Type of treatment in deteriorated patients: Zinc (50 mg daily) with or without DPA (no detailed data available) Additional risk factors of early neurological deterioration: Not analyzed	Not provided
Litwin et al. [35]	Retrospective analysis of 143 patients Definition: Any increase in UWDRS part II or an increase of ≥ 4 in part III or appearance of new neurological signs	Frequency of early neurological deterioration: Overall: 11.1% (16/143 patients) In neurological WD: 22.8% (16/70) In hepatic WD: 0% (0/73)	Type of treatment in deteriorated patients: DPA: 75% (12/16) Zinc sulfate: 25% (4/16) (NS vs DPA) Risk factors of early neurological deterioration: - Initial severity of neurological WD scored in UWDRS part II and III - Brain MRI lesions located in pons and thalamus - Concomitant treatment with drugs blocking dopamine neurotransmission	Persistent: 30.0% (5/15) (2 patients died) Partial recovery: 13.3% (2/15) Recovery: 53.3% (8/15) Lost to follow-up: 10.5% (1/16)
Kalita et al. [43]	Prospective study of 63 patients Definition: > 10% worsening in baseline Burke-Fahn-Marsden score or appearance of new neurological signs	Frequency of early neurological deterioration: Overall: 30.1% (19/63) In neurological WD: 32.2% (19/59) In presymptomatic individuals: 0% (0/4)	Type of treatment available only for all patients: DPA monotherapy: 38.0%% (24/63) DPA with zinc acetate: 47.6% (30/63) Zinc acetate monotherapy: 14.2% (9/63) Risk factors of early neurological deterioration: - Drooling - Leukopenia - Thrombocytopenia - Splenomegaly - Evidence of chronic liver disease	Persistent: 21.0% (4/19) Partial recovery: 52.6% (12/19) Recovery: 5.2% (1/19) Lost to follow-up: 10.5% (2/19) DPA was withdrawn if deteriorated. In 2 cases, TN was introduced (improvement); others were treated with zinc acetate (remained only or were switched from DPA)
Chang et al. [37]	Retrospective study of 65 patients Definition: Not provided	Frequency of early neurological deterioration: In neurological WD: 0% (0/21) In hepatic WD: 0% (0/44)	No cases of worsening. All patients were treated with DPA plus zinc sulfate Additional risk factors of early neurological deterioration: Not analyzed	Not applicable (no cases of worsening)
Weiss et al. [36]	Retrospective study of 405 patients Definition: Not provided (deterioration in physical examination)	Frequency of early neurological deterioration: Overall: 4.6% (19/405) In neurological WD: 13.1% (19/144) In hepatic WD: 0% (0/207) In asymptomatic individuals: 0% (0/54)	Type of treatment in deteriorated patients: DPA: 2% (6/295) as first line and 3.4% (1/31) as second-line treatment TN: 10.5% (4/38) as first line and 7.8% (8/103) as second-line treatment (NS vs DPA) Additional risk factors of early neurological deterioration: Not analyzed	Not provided
Weiss et al. [42]	Retrospective study of 288 patients Definition: Not provided (neurological assessment)	Frequency of early neurological deterioration: Overall: 14.9% (43/288) No data by phenotype	Type of treatment in deteriorated patients: DPA: 9.1% (22/243) TN: 8.8% (9/102) ZS: 9.5% (9/95); used only as second-line treatment in 65 patients Combination therapy: 7.3% (3/41) Additional risk factors of early neurological deterioration: Not analyzed	Not provided
Linn et al. [38]	Retrospective study of 17 patients Definition: Not provided (assessment in Rankin scale at end of follow-up)	Frequency of early neurological deterioration: Overall: 0% (0/17) In neurological WD: 0% (0/10) In hepatic WD: 0% (0/7)	All patients treated with zinc sulfate Additional risk factors of early neurological deterioration: Not analyzed	Not applicable (no cases of worsening)
Brewer et al. [41]	Prospective study of 48 patients Definition: Not provided (neurological assessment in neurological score and speech score)	Frequency of early neurological deterioration: In neurological WD (all patients): 14.5% (7/48)	Type of treatment in deteriorated patients: TN plus ZS: 26.0% (6/23) Tetrathiomolybdate plus ZS: 4.0% (1/25) Additional risk factors of early neurological deterioration: Not analyzed	Persistent: 100% (7/7) 3 patients on TN died due to complications of immobilization
Medici et al. [40]	Prospective study of 35 patients Definition: Not provided (assessment in neurological scale 0–33 points)	Frequency of early neurological deterioration: Overall: 17.1% (6/35) In neurological WD: 50% (6/12) In hepatic WD: 0% (0/23)	Type of treatment in deteriorated patients: DPA: 26% (6/23) Zinc sulfate: 0% (12/12) Additional risk factors of early neurological deterioration: Not analyzed	Persistent: 16.7% (1/6) Complete recovery: 83.3% (5/6)
Marcellini et al. [39]	Prospective study of 22 patients Definition: Not provided (assessment by neurologist)	Frequency of early neurological deterioration: Presymptomatic individuals: 0% (0/22)	All patients treated with zinc sulfate Additional risk factors of early neurological deterioration: Not analyzed	Not applicable (no cases of worsening)
Brewer et al. [44]	Prospective study of 55 patients Definition: Not provided (assessment by neurologist, neurological score and speech score)	Frequency of early neurological deterioration: In neurological WD (all patients): 3.6% (2/55)	Type of treatment in deteriorated patients: Tetrathiomolybdate plus ZS: 3.6% (2/55) Additional risk factors of early neurological deterioration: Not analyzed	Not provided

*DMPS*, dimercaptopropane sulfonic acid; *DPA*, d-penicillamine; *GAS for WD*, Global Assessment Scale for Wilson’s Disease; *IV*, intravenous; *NS*, not significant between treatment types; *sNfL*, serum neurofilament light chain; *TN*, trientine; *UHDRS*, Unified Huntington Disease Rating Scale; *UWDRS*, Unified Wilson Disease Rating Scale; *WD*, Wilson’s disease; *ZS*, zinc salts

**Table 2:** Summary of case reports analyzing the early neurological deterioration in WD patients according to their initial phenotype of WD, anti-copper treatment, potential risk factors of neurological deterioration as well as outcome of deterioration

Reference	Patient characteristics	Type of WD treatment in deteriorated patients and any information on risk factors for deterioration	Outcome
Czlonkowska et al. [26]	21-year-old man with hepatic WD (liver cirrhosis with portal hypertension) Brain MRI presented changes in the basal ganglia	DPA 750 mg/day with ZS 180 mg/day elementary zinc After 1 month — rigidity, dysarthria, drooling, next severe extrapyramidal syndrome occurred Risk factors of early neurological deterioration: DPA introduced too fast (750 mg as initial dose with ZS)	Not recovered
Dusek et al. [49]	27-year-old man with neurological WD (cervical dystonia, ataxia, dysarthria and action tremor of both hands; UWDRS total score 30 points)	DPA slowly introduced up to 450 mg daily within 5 months After DPA initiation hand ataxia and ataxia progressed; gait and posture instability and leg dystonia newly developed during 5 months (UWDRS 60 points at month 5) Risk factors of early neurological deterioration: not found	Not recovered
Dziezyc et al. [25]	35-year-old woman with neurological WD (tremor; UWDRS part II and III 7 points)	ZS 180 mg/day elementary zinc Worsening progressive after 6 months — whole body dystonia and rigidity (UWDRS part II and III 54 points) Risk factors of early neurological deterioration: Not analyzed	Not recovered
Berger et al. [48]	38-year-old woman with neurological WD (postural tremor of both hands, depression) and liver injury (elevated liver transaminases) Brain MRI: ponto-mesencephalo-thalamic lesions	DPA introduced slowly 150 mg/day Epileptic status occurred Risk factors of early neurological deterioration: Not analyzed	Recovered
Kim et al. [13]	21-year-old man with neurological WD (dystonia and parkinsonism)	TN 1000 mg/day increasing to 1500 mg/day over 3 months Worsening after 4 months — whole body dystonia, altered mental status Risk factors of early neurological deterioration: Not analyzed	Not recovered (18 months follow-up)
Kleinig et al. [12]	32-year-old woman with neurological WD (mild parkinsonism) with liver cirrhosis	DPA 1500 mg/day Worsening after 3 months — dystonia Risk factors of early neurological deterioration: Not analyzed	Not provided
Sohtaoglu et al. [47]	77-year-old woman with neurological WD (mild head tremor, slight forgetfulness, dysarthria and kinetic tremor in arms) Brain MRI: presented bilateral changes in basal ganglia	DPA slowly introduced and escalated to 900 mg/day with zinc 150 mg/day Worsening after 2 weeks severe parkinsonism, jaw-closing dystonia, patient became bedridden, Brain MRI follow-up: more prominent changes in basal ganglia and white matter involvement Risk factors of early neurological deterioration: Not analyzed	Not recovered (deceased due to cardiopulmonary arrest)
Paul et al. [20]	8-year-old girl with hepatic WD (liver function tests elevated)	DPA (dose not known) Extrapyramidal symptoms occurred Risk factors of early neurological deterioration: Not analyzed	Recovered
Brewer [24]	27-year-old woman with neurological WD (slight tremor; depression)	DPA 1000 mg/day Severe worsening after 4–8 weeks — general dystonia, dysarthria, dysphagia Risk factors of early neurological deterioration: DPA introduced at a high dose	Not recovered
Porzio et al. [19]	9-year-old boy with hepatic WD (liver function tests elevated for 2 years)	DPA 600 mg/day Rigidity and tremor occurred in 10 weeks — switched to zinc Risk factors of early neurological deterioration: DPA introduced at full dose	Recovered
Walshe and Munro [23]	17-year-old woman with neurological WD (slight tremor)	ZS (dose not known) Worsening at 6 months — parkinsonism, tremor, dysarthria, contractures Risk factors of early neurological deterioration: Not analyzed	Recovered partially
Brewer et al. [18]	19-year-old man with hepatic WD (liver function tests elevated). Patient was not treated for 2 years (was diagnosed at age 17)	DPA (dose not known) Severe worsening — dysarthria, dysphagia, drooling, rigidity and tremor Risk factors of early neurological deterioration: Not analyzed	Not recovered
Veen et al. [22]	30-year-old woman with neurological WD (slight tremor, chorea, myoclonus with liver disease, increased liver functions tests)	DPA 375 mg/day (slowly introduced) Severe worsening after 9 days — general somnolence, tremor (drug replaced by zinc) Risk factors of early neurological deterioration: Not analyzed	Recovered
Hilz et al. [16]	31-year-old man with neurological WD (tremor)	DPA 1 g IV four times daily Severe worsening — akinesia, mutism, respiratory insufficiency, improvement seen after DPA dose reduction Risk factors of early neurological deterioration: DPA introduced at a high dose	Partially reversible
Glass et al. [17]	28-year-old man with hepatic WD (liver cirrhosis). Brain MRI presented changes in the basal ganglia and brain atrophy	DPA 1500 mg/day Severe worsening — dysarthria, dysphagia, drooling, dystonia and psychiatric symptoms Risk factors of early neurological deterioration: DPA introduced at full dose	Partially recovered
Brewer et al. [15]	19-year-old man with neurological WD (slight tremor, emotional outburst)	DPA 1000 mg/day (full dose) Severe worsening after 2 weeks — general dystonia, dysarthria, dysphagia, bedridden Risk factors of early neurological deterioration: DPA introduced at full dose	Partially recovered

*DPA*, d-penicillamine; *IV*, intravenous; *TN*, trientine; *UWDRS*, Unified Wilson Disease Rating Scale; *WD*, Wilson’s disease; *ZS*, zinc salts

**Fig. 2:**
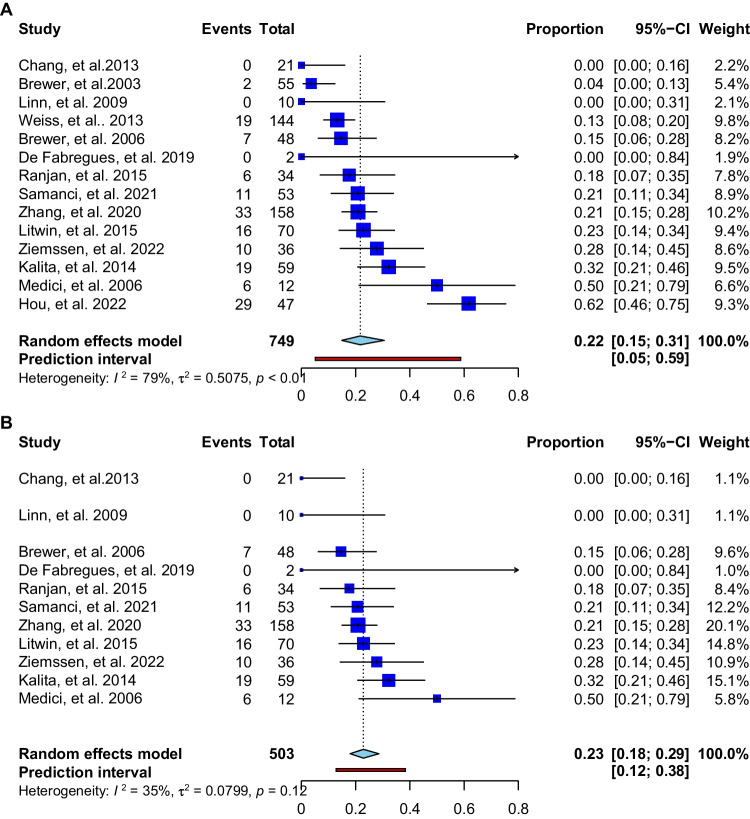
(**A**) Meta-analysis available studies (apart from case reports) showing frequency of early neurological deterioration in WD patients with neurological symptoms; (**B**) meta-analysis of available studies showing the frequency of early neurological deterioration in WD patients with neurological symptoms after removing the studies with the greatest contribution to heterogeneity

The 32 publications included 1512 WD patients in whom 217 cases of early neurological deterioration were described, indicating a frequency of 14.3%. In available analysis (excluding the papers without detailed phenotypic presentation) [42], the early neurological deterioration occurred mostly in patients with the neurological phenotype (21.8% [167/763]; see Fig. 2A shows studies in WD patients with neurological symptoms; Fig. 2B shows studies after the exclusion of studies with the greatest contribution to heterogeneity [28, 36, 44] see Supplementary Fig. 1). Deteriorations occurred very rarely in hepatic cases (1.3% [5/377]) and never in 87 asymptomatic individuals. Most deteriorations were described in patients treated with DPA: 70.5% (153/217). Less frequently, early neurological deterioration occurred in patients receiving TN (14.2% [31/217]), ZS (6.9% [15/217]), DMPS and zinc (5.0% [11/217]); molybdate (1.4% [3/217]) and 1.8% (4/217) on combined therapy with ZS and chelators.

The main risk factor for neurological deterioration in WD patients was neurological phenotype (21.8% risk vs 1.3% risk in hepatic WD phenotype). Other risk factor was the initial high dose treatment with high DPA (7/16 (43.7%) reported in case reports [12, 15–17, 19, 24, 26]. Other well-documented risk factors of early neurological deterioration were (1) initial severity of neurological disease WD scored in clinical scales, in brain magnetic resonance imaging (MRI) semiquantitative scale (or lesions in pons), as initial serum concentration of neurofilaments (sNfL) [27]; (2) severity of liver disease [45] or (3) concomitant drugs blocking dopaminergic neurotransmission [35].

Data regarding patients’ recovery was provided for 128 WD patients; 24.2% (31/128) completely recovered, 27.3% (35/128) recovered partially, 39.8% (51/128) did not improve and 11 patients were lost to follow-up.

## Discussion

This is the first systematic review of the literature focused on early neurological deterioration in WD patients. Based on our previous papers [27, 35], we defined early neurological deterioration as worsening up to 6 months of anti-copper treatment initiation to verify the available results and present more homogenous data. We documented that early neurological deterioration in WD occurred in about 14% of all WD patients and 21.8% of patients with neurological symptom. These data show that this phenomenon may currently occur less frequently than reported previously, particularly in some older review studies, which cited frequency up to 50% [15–18, 24]. A potential reason for this difference is that DPA was the first drug introduced as a WD treatment and initial reports of early neurological deterioration were mostly describing DPA-treated patients. The longest experience with WD treatment as well as adverse drug reactions relates to DPA and next to chelators (mostly used to treat symptomatic WD patients), which affected the assessment of the frequency of neurological deterioration. Since the link between early neurological deterioration and full-dose DPA was made, clinicians have adopted a “start low and go slow” approach to dosing, which has contributed to decreased frequency. Moreover, current WD treatment recommendations emphasize the DPA dose titration during treatment initiation [1]. However, retrospective studies and national registries still included older cases in analysis [4–8], which could have affected our results. But due to mostly summarized results of these studies, the separate analysis of such cases (apart from case reports [12, 15–17, 19, 24, 26] were not available.

It is noteworthy that the most often cited article reporting the high frequency of neurological deterioration after DPA was published in 1987 [15] and was based on a questionnaire filled in by patients or their families. Out of 54 patients, 28 completed the questionnaire and 3 patients did not have neurological presentation, so only 25 patients were analyzed (45% of initial group), of which neurological worsening occurred in 13 (52%) patients. However, in 3 cases, worsening occurred between 6 and 12 months after DPA introduction and after re-analyzing their results, they calculated a frequency of 18.5% for neurological deteriorations (10/54), not using objective neurological scales.

Our study additionally verified the irreversibility of early neurological deteriorations. Based on our systematic review, we found complete or partial recovery in around half of the patients, which is higher than previously reported [35], but the lack of improvement in other patients highlights the severe nature of the phenomenon. Indeed, these neurological complications have contributed to the search for new anti-copper drugs, such as TN or molybdate salts.

Additionally, we found that most early neurological deteriorations occurred in WD patients initially diagnosed with the neurological phenotype of the disease. Ziemssen et al. [27] found that early neurological deterioration was related to sNfL levels (as a marker of severity of neuroaxonal injury), the severity of neurological disease scored on the Unified Wilson Disease Rating Scale (UWDRS) and the chronic damage score subscale in brain semiquantitative MRI scale [27]. The semiquantitative brain MRI scale in WD consists of an acute damage score (which reflects oedema, demyelination — potentially acute — “fresh” reversible changes) and a chronic damage score (which reflects necrosis, brain atrophy as well as iron accumulation secondary to neuronal necrosis — the irreversible changes) [27]. The finding that only the chronic damage score predicts neurological worsening may suggest that in some patients who neurologically deteriorate, there are irreversible advanced neurodegenerative processes that cannot be stopped by drugs — they present with natural progression and not early neurological deterioration.

Other, proposed in the available literature, risk factors for early neurological worsening include the use of concomitant drugs blocking dopaminergic neurotransmission, lesions located in the pons and thalamus, which again indicate advanced neurological disease [35], and advanced liver disease (e.g.. evidence of chronic liver disease, splenomegaly, leukopenia, thrombocytopenia and drooling) [43].

Taken together, these observations indicate a need to distinguish the natural course of the disease from treatment-related early neurological deteriorations and additionally lead to discussions about how to establish the diagnosis and a definition of early neurological deterioration in neurologically symptomatic patients, which are currently lacking. As part of these studies, the so-called “therapeutic lag” effect [50] could be investigated, which is seen in other neurological disorders, like multiple sclerosis. After the introduction of anti-copper drugs in WD, the expected time to effects on liver function tests and liver symptoms is 4–6 months, but data on neurological symptoms are more limited and they may take longer to improve [1]. It would be interesting to conduct studies in WD to establish the expected time course of neurological symptoms regression, including initial brain MRI changes as well as biomarkers of nervous system injury [4, 27, 45].

### Study limitations

Our study has some limitations. Studies were heterogenous, particularly regarding the treatment. In line with current recommendations [1] most of the symptomatic WD patients were treated with chelators (mostly DPA). Therefore, we were not able to conduct a meta-analyses comparing deterioration risk between different treatments regarding the type of anti-copper treatment and neurological deterioration. Some of the studies included only [28, 44] neurological or mostly hepatological WD patients [36]. The meta-analysis was limited by substantial heterogeneity, but the estimate was stable after excluding the studies with the greatest contribution to heterogeneity [28,36.44] (Supplementary Fig. 1 and Fig. 2B). Further, some studies included patients switched from other anti-copper drugs and/or receiving combination treatments. We tried to control for this with our inclusion criteria. Additionally, the definition of neurological worsening differed, although we tried to standardize this to a degree with our inclusion of cases within the first 6 months of treatment. Details were missing for some cases of neurological worsening in retrospective WD long-term follow-up studies and these were not included [6–8, 29, 51–65].

## Conclusions

The early neurological deteriorations in WD occur less frequently than previously reported; however, it is still present in around 14% of WD patients and almost one quarter of patients with initial neurological phenotype of WD. Further studies are required to distinguish the natural progression of the disease from an early neurological deterioration, including considerations of “therapeutic lag” [47] as well as brain MRI studies [66], and biomarkers of central nervous system involvement [66]. Results of such studies may help develop an evidence-based definition for an early neurological deterioration.

## Supplementary information

Below is the link to the electronic supplementary material.

Supplementary file1 (PDF 7 KB)
