# Supplementary figures and images for: Early neurological deterioration in Wilson’s disease: a systematic literature review and meta-analysis
Source: Neurol Sci. 2023 Jun 14;44(10):3443–55. doi: 10.1007/s10072-023-06895-6 (PMC10495500; doi:10.1007/s10072-023-06895-6)

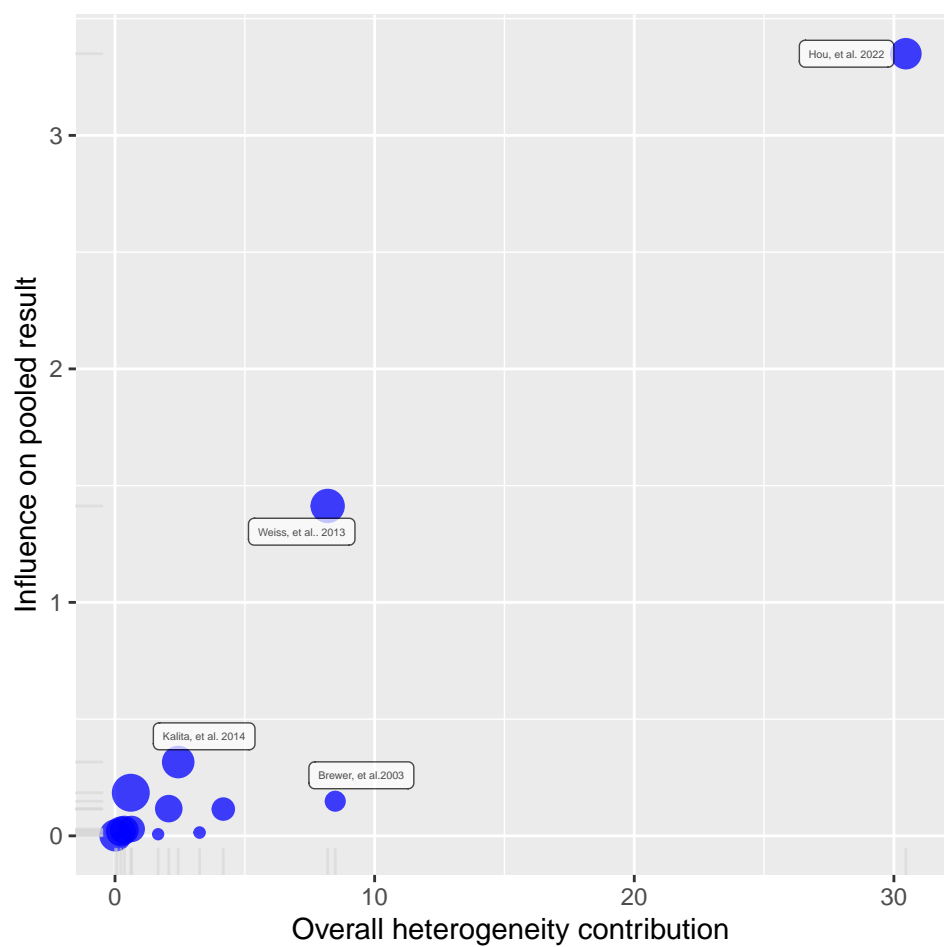

Supplement: Supplementary file 1 — Supplementary file1 (PDF 7 KB) [file 10072_2023_6895_MOESM1_ESM.pdf]
